# Robust dynamic experiments for the precise estimation of respiration and fermentation parameters of fruit and vegetables

**DOI:** 10.1371/journal.pcbi.1009610

**Published:** 2022-01-12

**Authors:** Arno Strouwen, Bart M. Nicolaï, Peter Goos

**Affiliations:** 1 Department of Biosystems, KU Leuven, Leuven, Belgium; 2 Department of Engineering Management, University of Antwerp, Antwerp, Belgium; University of Connecticut School of Medicine, UNITED STATES

## Abstract

Dynamic models based on non-linear differential equations are increasingly being used in many biological applications. Highly informative dynamic experiments are valuable for the identification of these dynamic models. The storage of fresh fruit and vegetables is one such application where dynamic experimentation is gaining momentum. In this paper, we construct optimal O_2_ and CO_2_ gas input profiles to estimate the respiration and fermentation kinetics of pear fruit. The optimal input profiles, however, depend on the true values of the respiration and fermentation parameters. Locally optimal design of input profiles, which uses a single initial guess for the parameters, is the traditional method to deal with this issue. This method, however, is very sensitive to the initial values selected for the model parameters. Therefore, we present a robust experimental design approach that can handle uncertainty on the model parameters.

## Author summary

Fruit and vegetables need to be stored at low temperature and oxygen conditions as well as slightly heightened carbon dioxide conditions so that they remain fresh throughout the entire year. The exact storage conditions are different for each cultivated variety. Optimizing these storage conditions typically requires a lot of experimentation. Traditionally, this was done by independently storing the fruit of vegetable product at many different combinations of temperature as well as oxygen and carbon dioxide conditions, and by tracking the quality of the product and choosing the best of these conditions. This, however, is a very wasteful approach as the quality tracking at certain conditions do not inform us about the quality at different storage conditions. Instead, we adopt a model-based approach, where the product is described as a dynamic system with inputs and outputs. This model has parameters that must be estimated from experimental data. But once the model has been calibrated it can be used to make predictions at any storage condition. We develop the experimental design methodology required to precisely estimate the model parameters. We do this in a robust manner, meaning we are able to discover the true model parameter values no matter their specific value.

## Introduction

Model-based approaches are commonly used in the analysis, control and optimization of biological systems. These models rely on knowledge of physical, chemical and biological laws, such as mass balances, transport phenomena and reaction kinetics, and are often described by a system of non-linear differential equations, with inputs and outputs. So, often, the structure of a model can be determined from first principles. However, the model will generally also rely on parameters whose numerical values cannot be determined from physical laws. These parameters must then be inferred from experimental data before the model can be put to use. The experiments required to estimate these parameters are often laborious and cost prohibitive. It is therefore important to determine experimental conditions which are rich in information and thus allow a precise estimation of the unknown model parameters.

At present, the model parameters are often estimated from data collected using multiple experiments at various combinations of input levels, which are kept constant throughout each individual experiment. Even if an appropriate experimental design technique is used to reduce the number of experiments that have to be performed, the experimental effort remains considerable. Alternatively, experiments in which the inputs vary in time can be conducted. This has been shown to be a cost-effective way to generate a highly informative data set [1]. Such experiments are called dynamic. In optimal design of dynamic experiments, time-varying input profiles are constructed to optimize the information content of a single experiment.

The major challenge for experimental design for any non-linear model is the dependence of the optimal experiment on the true values of the unknown model parameters, whose estimation is the primary goal of the experiment. Most of the experimental design literature uses a scalar metric of the Fisher information matrix as the measure of information content in an experiment, as this matrix is inversely related to the covariance matrix of the model parameters. An informative experiment thus ensures a small covariance matrix of the model parameters. Locally optimal design of input profiles uses initial guesses for the model parameters to calculate this information matrix [2]. This method has already been used to construct informative time-varying inputs in chemical engineering [3] and in biological fields such as systems biology [4], predictive microbiology [5, 6], food engineering [7, 8] and synthetic biology [9]. However, an input profile that is highly informative for one set of parameter values may lack information for other parameter values. Thus, if the initial guesses of the parameters differ substantially from the true values, then the locally optimal design might not allow precise parameter estimates. So, locally optimal design is sensitive to the initial parameter guesses and is thus not robust.

Much recent research in experimental design for non-linear models aims at robustifying the design to the true, but unknown, values of the model parameters. A robust design provides a large information content regardless of the true values of these parameters. For dynamic experiments, in particular, a min-max based approach has been used by [10, 11]. Here, the design is optimized for a worst case scenario. Fisher information matrices are calculated for a set of possible parameter values and the experiment is scored based on the least informative matrix in this set. In contrast, an expected value approach is taken by [12, 13]. In this approach, the average information content of the experiments over all possible parameter values is optimized. The expected value approach tends to perform better than the min-max approach for a large subset of probable parameter values, but not for extreme sets of parameter values. The expected value approach is also called pseudo-Bayesian experimental design, because the possible parameter values can be expressed using a prior distribution [14]. The expected value approaches of [12, 13] rely on parametric distributions to describe the uncertainty about the model parameters before the experiment has taken place. However, for non-linear models, a parametric distribution will often not be appropriate to quantify the uncertainty about the model parameters. Therefore, in this work, we allow arbitrary distributions to quantify the model parameter uncertainty. More specifically, we show how the results of a Bayesian analysis of historical data using Markov-chain Monte-Carlo can be used as a prior distribution, when optimizing an experimental design. This Markov-chain can then be used to calculate the average Fisher information matrix, and has the advantage that it can represent arbitrary distributions [15].

Post-harvest storage of fresh fruit and vegetables is one biological application where optimal experimental design is useful. The ideal storage temperature as well as O_2_ and CO_2_ partial pressures depend on the respiration characteristics, which in turn depend on species, cultivar, ripeness and multiple other factors. Determining the ideal storage conditions therefore requires much experimentation. Traditionally, this was done by independently storing the product at many different combinations of temperature as well as O_2_ and CO_2_ partial pressures, and by monitoring the respiration and fermentation [16]. Many modern storage applications, such as modified atmosphere packaging [17] and dynamic controlled atmosphere [18], adopt a model-based approach, where the product is described as a dynamic system with inputs and outputs. The resulting models enable us to use the tools of optimal dynamic experimental design to construct informative experiments. The respiration and fermentation kinetics are generally described by a model of the Michaelis-Menten type [19]. Robust experimental design is particularly needed for post-harvest applications because of the large biological variability of fresh fruit and vegetables. As a consequence of that variability, the parameters of the aforementioned kinetic models vary substantially between seasons and origins. In this paper, we therefore focus on constructing robust experimental techniques to estimate the respiration and fermentation kinetics of pear fruit. This paper describes the first use of robust optimal experimental design techniques in post-harvest research.

## Robust optimal experimental design for dynamical systems

In this section, we first present the type of dynamical models considered in this paper. Then, we show how to quantify the information gained from measurements, using the Fisher information matrix. Next, we discuss how to maximize this information content using appropriate control inputs. Finally, we explain how to make the optimal control inputs robust.

### Dynamic models

In this paper, we consider experimental design for dynamic models of the form:

$$\begin{array}{c} \begin{array}{cc} \frac{\text{d}x}{\text{d}t} & =f\left(t,x,\theta ,u\left(t\right)\right),\;\text{with}\;x\left(t=0\right)=x_{0};\\ y_{k} & =h\left(x\left(t_{k}\right)\right)+\epsilon_{k}, \end{array} \end{array}$$ (1)

where *t* denotes the time ranging from 0 to *t*_*e*_, the end time of the experiment. The column vector ***y***_*k*_ contains the measurements taken at time point *t*_*k*_, with *k* ranging from 1 to *N*, the number of measurement times. The time between measurements is equally spread, so that *t*_*k*_ = *kt*_*e*_/*N*. A measurement at the end of the experiment is thus included, but not at the start. The measurements are subject to independent Gaussian noise. More specifically, ***ϵ***_*k*_ is identically and independently multivariate normally distributed with zero mean and covariance matrix *R*(***y***), which may depend on the true value of the measurements, uncorrupted by noise. The measurements depend on the dynamic state column vector ***x***(*t*) through the measurement function *h*. The states ***x***(*t*) have to be calculated from the system of ordinary differential equations *f*, with initial conditions ***x***_0_. This system depends on the unknown model parameter column vector ***θ***, and the controllable input column vector ***u***(*t*).

### Information content of an experiment

Our goal is to optimize the controllable inputs ***u***(*t*) so that the measurements ***y***_*k*_ contain as much information as possible about the unknown parameters ***θ***. A popular way to quantify the information content of an experiment is the Fisher information matrix (FIM) [2, 21]. The (m,n)th entry of this matrix is given by:

$$\begin{array}{c} \begin{array}{cc} F_{m,n}\left(\theta ,u\left(t\right)\right)= & \;\sum_{k=1}^{N}({\frac{\partial x(t_{k})}{\partial \theta_{m}}}^{T}{\frac{\partial h}{\partial x}}^{T}R^{-1}\left(y\right)\frac{\partial h}{\partial x}\frac{\partial x(t_{k})}{\partial \theta_{n}}+\\ & \frac{1}{2}\text{Tr}(R^{-1}\left(y\right)\sum_{i=1}^{n_{y}}(\frac{\partial R}{\partial y_{i}}\frac{\partial h_{i}}{\partial x}\frac{\partial x(t_{k})}{\partial \theta_{m}})R^{-1}\left(y\right)\sum_{i=1}^{n_{y}}(\frac{\partial R}{\partial y_{i}}\frac{\partial h_{i}}{\partial x}\frac{\partial x(t_{k})}{\partial \theta_{n}}))), \end{array} \end{array}$$ (2)

with *n*_*y*_ the measured output dimension. The sensitivities of the states to the unknown parameters, *∂****x***(*t*_*k*_)/*∂****θ***, cannot be computed directly, as the evolution over time of the states ***x***(*t*) is described by the system of differential equations in Eq (1). However, these sensitivities can be calculated from the forward sensitivity differential equations [22]:

$$\begin{array}{c} \frac{\text{d}}{\text{d}t}\frac{\partial x}{\partial \theta }=\frac{\partial }{\partial \theta }\frac{\text{d}x}{\text{d}t}=\frac{\partial f}{\partial x}\frac{\partial x}{\partial \theta }+\frac{\partial f}{\partial \theta },\;\text{with}\;\frac{\partial x(t=0)}{\partial \theta }=0. \end{array}$$ (3)

The FIM is the inverse of the Cramer-Rao lower bound of the variance of an unbiased estimator of ***θ***. This lower bound can be interpreted geometrically as a hyper-ellipsoid defined by the eigenvectors and the inverse of the eigenvalues of the FIM. We want this lower bound to be as small as possible, and thus the eigenvalues of the FIM to be as large as possible, because this implies precise estimates for ***θ*** are possible. In the literature, several scalar functions of the eigenvalues are used to quantify the size of the FIM [23]. To this end, we use the product of the eigenvalues which equals the determinant of the FIM, and is thus also called the D-optimality criterion. This criterion is inversely related to the volume of the hyper-ellipsoids, measuring the uncertainty about the parameter vector ***θ***.

### Discretizing the controls

Optimal experimental design for dynamic systems is an infinite dimensional optimization problem since it requires finding optimal controls ***u***(*t*) for every *t* ∈ [0, *t*_*e*_]. To make this problem tractable, the controls have to be discretized in time. We utilize a bounded piecewise constant discretization allowing ***u***(*t*) to switch values at *M* equally spaced time points,

umin≤u(t)=∑j=1Mujχ[(j-1)teM,jteM[(t)≤umax, (4)

where ***u***_*j*_ is the constant control vector during the interval [(j-1)teM,jteM[, and *χ*_*A*_ is the indicator function,

$$\begin{array}{c} \chi_{A}\left(t\right)=\{\begin{array}{c} 1\;t\in A\\ 0\;t\notin A, \end{array} \end{array}$$ (5)

and ***u***_min_ and ***u***_max_ are the minimum and maximum control values allowed. Piecewise constant input profiles do not only have the benefit of making our optimization problem tractable, but they are also easy to implement in practice.

### Robustifying the experiment

Another issue with experimental design for models that are non-linear in the parameter vector ***θ***, such as the model we described in Eq (1), is the dependence of the FIM on ***θ***. This presents us with a cyclic problem because we are performing the experiment to quantify those parameters. Locally optimal experimental design is the traditional method to deal with this issue. In this approach, the FIM is calculated using a single initial guess θ* obtained from available prior knowledge. A locally optimal experimental design is thus given by:

$$\begin{array}{c} \underset{u_{1}\ldots u_{M}}{\text{arg max}}\;|F(\theta^{*},u\left(t\right))|\;\text{subject}\;\text{to}\;u_{\text{min}}\leq u\left(t\right)\leq u_{\text{max}}. \end{array}$$ (6)

This method might give poor results if the initial guess deviates from the true value, and is thus not robust. One method to robustify the optimal experimental design can be achieved by averaging the information content of the experiment over multiple possible values of the parameters, taking into account the likelihood of each parameter value. More specifically, a weighted average is used to quantify the information content, where the weights are given by a prior distribution of the parameters *p*(***θ***). This distribution represents the belief of uncertainty concerning these parameters, before the experiment has been performed. A robust optimal experimental design is therefore given by:

$$\begin{array}{c} \underset{u_{1}\ldots u_{M}}{\text{arg max}}\;\int |F(\theta ,u(t))|p\left(\theta \right)\text{d}\theta \;\text{subject}\;\text{to}\;u_{\text{min}}\leq u\left(t\right)\leq u_{\text{max}}. \end{array}$$ (7)

This criterion is also called the pseudo-Bayesian D-optimality criterion, because it uses a prior distribution, a tool from Bayesian statistics, in combination with the Fisher information matrix, a tool from classical frequentist statistics. Replacing the Fisher information matrix with the Bayesian information matrix [15] and the determinant with a log-determinant would lead to the fully Bayesian and decision theory based approach of [14].

Generally, the integral in Eq (7) cannot be evaluated analytically. To approximate it numerically, we draw *R* random model parameter vectors, ***θ***_*r*_, from the prior distribution *p*(***θ***) and average the determinant of the FIM over these values:

$$\begin{array}{c} \;\int |F(\theta ,u(t))|p\left(\theta \right)\text{d}\theta \approx \frac{1}{R}\sum_{r=1}^{R}|F(\theta_{r},u\left(t\right))|\;\theta_{r}∼p\left(\theta \right). \end{array}$$ (8)

The robust criterion in Eq (7) is thus calculated by averaging the determinant of the FIM over a sample drawn from the prior distribution.

### Numerical details

The entire optimization problem was implemented in the Julia programming language [24]. All differential equations were solved using the Tsitouras 5/4 Runge-Kutta method [25], as implemented in OrdinaryDiffEq.jl [26], with relative and absolute tolerances equal to 1 × 10^−3^ and 1 × 10^−6^, respectively. The piecewise constant control switches were implemented using a periodic callback, provided by DiffEqCallbacks.jl. Note that ***u***(*t*_*e*_) in Eq (4) is undefined, but this value does not influence the FIM.

Because coding the sensitivity differential equations in Eq (3) by hand is quite laborious and error prone, we calculated them exploiting the automatic differentiation capabilities of DiffEqSensitivity.jl [27], more specifically its implementation of the discrete forward sensitivity analysis method.

To solve the non-linear optimization problems, we used the box constrained optimization capabilities of NLopt.jl [28], specifically the method of moving asymptotes [29], with a relative tolerance on the objective function of 1 × 10^−3^. The required gradients for this method are calculated using the nested differentiation capabilities of ForwardDiff.jl [30]. This optimization method requires an initial experimental design to improve upon and is a local optimizer. Thus, it is not guaranteed to find the absolute best experimental design. To deal with this issue, we utilize multiple starts, each with a different initial design. The TikTak global optimization algorithm [31] can carefully select these initial designs from the design space, using Sobol points. We use 1000 starts of the interior point optimization method in combination with the TikTak implementation in MultiStartOptimization.jl.

## Respiration and fermentation model of pear fruit

In this paper, we apply our robust experimental design methodology to precisely estimate the respiration and fermentation characteristics of pear fruit. First, we present a respiration and fermentation model of pear fruit inside a jar. We then quantify the initial uncertainty concerning the various parameters in this model using a published data set.

### Model description

The respiration and fermentation of pear fruit inside a jar is modeled by two mass balances for O_2_ and CO_2_:

$$\begin{array}{c} \begin{array}{cc} V_{\text{j}}\frac{d\left[{\text{O}}_{2}\right]}{dt} & =Q_{\text{in}}\left(t\right){\left[{\text{O}}_{2}\right]}_{\text{in}}\left(t\right)-Q_{\text{out}}\left(t\right)\left[{\text{O}}_{2}\right]-m_{\text{p}}r_{{\text{O}}_{2}}\left(t\right),\\ V_{\text{j}}\frac{d\left[{\text{CO}}_{2}\right]}{dt} & =Q_{\text{in}}\left(t\right){\left[{\text{CO}}_{2}\right]}_{\text{in}}\left(t\right)-Q_{\text{out}}\left(t\right)\left[{\text{CO}}_{2}\right]+m_{\text{p}}r_{{\text{CO}}_{2}}\left(t\right). \end{array} \end{array}$$ (9)

**Fig 1:**
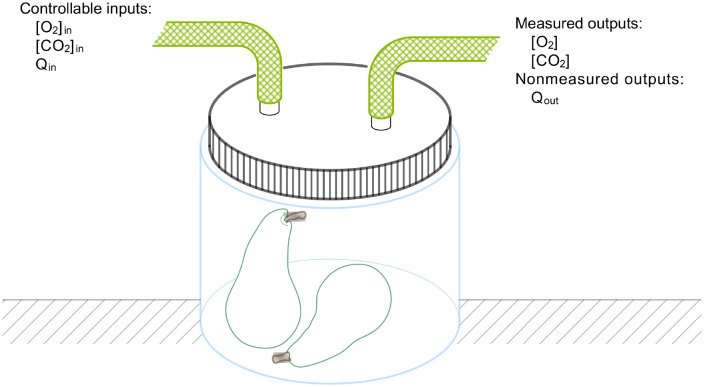
Schematic representation of the measurement setup.

The square brackets in these expressions represent concentrations in mol/m^3^. These differential equations describe the change of O_2_ and CO_2_ concentrations inside a jar with volume *V*_j_, which equals 5 dm^3^ in our examples. A time varying air mixture with an oxygen concentration [O_2_]_in_(*t*) and a carbon dioxide concentration [CO_2_]_in_(*t*) is blown into the jar with flow rate *Q*_in_(*t*) (units: m^3^/h). These three time varying functions form the controllable inputs to our system. Our measurement set up is schematically shown in Fig 1.

Since the pressure inside the jar should remain equal to the atmospheric pressure, we can calculate the outflow *Q*_out_(*t*) from the jar using the ideal gas law:

$$\begin{array}{c} Q_{\text{out}}\left(t\right)\frac{P_{\text{atm}}}{\bar{R}T}=Q_{\text{in}}\left(t\right)\frac{P_{\text{atm}}}{\bar{R}T}-m_{\text{p}}r_{{\text{O}}_{2}}\left(t\right)+m_{\text{p}}r_{{\text{CO}}_{2}}\left(t\right), \end{array}$$ (10)

with $\bar{R}$ the universal gas constant, *P*_atm_ the atmospheric pressure. Concentrations throughout the jar and at the outlet are considered to be similar, due to the assumption of well mixing. In our constructed experiments the temperature equals 293.15 K, the amount of O_2_ consumed and CO_2_ produced is proportional to the mass of the pears *m*_p_, taken to be 4 kg and the initial conditions for O_2_ and CO_2_ will be equal to regular air.

The respiration rates in Eq (10) are specified using models of the Michaelis-Menten type [19]:

$$\begin{array}{c} \begin{array}{cc} r_{{\text{O}}_{2}}\left(t\right) & =\frac{V_{\text{m},{\text{O}}_{2}}\left[{\text{O}}_{2}\right]}{\left(K_{\text{m},{\text{O}}_{2}}+\left[{\text{O}}_{2}\right]\right)\left(1+\frac{\left[{\text{CO}}_{2}\right]}{K_{\text{mn},{\text{CO}}_{2}}}\right)},\\ r_{{\text{CO}}_{2}}\left(t\right) & ={\text{r}}_{\text{q}}r_{{\text{O}}_{2}}\left(t\right)+\frac{V_{\text{m,f,}{\text{CO}}_{2}}}{1+\frac{\left[{\text{O}}_{2}\right]}{K_{\text{m,f},{\text{O}}_{2}}}}. \end{array} \end{array}$$ (11)

The models for these respiration rates contain six parameters that have to be identified. $V_{\text{m},{\text{O}}_{2}}$ and $V_{\text{m,f,}{\text{CO}}_{2}}$ are the maximum respiration and fermentation reaction rates, respectively. The Michaelis-Menten constant $K_{\text{m},{\text{O}}_{2}}$ represents the saturation of respiration at high oxygen levels, whereas $K_{\text{mn},{\text{CO}}_{2}}$ models the inhibition of respiration by CO_2_. The respiration quotient r_q_ represents the percentage of O_2_ that is converted to CO_2_ by respiration. Finally, $K_{\text{m,f},{\text{O}}_{2}}$ models the inhibition of fermentation by O_2_.

The measured gas concentrations [O_2_]_*m*_ and [CO_2_]_*m*_ at time point *t*_*k*_ are assumed to be equal to the true concentrations plus the additive Gaussian noise terms *ζ*_*k*_ and *η*_*k*_, respectively:

$$\begin{array}{c} \begin{array}{cc} {}[{\text{O}}_{2}{]}_{m}\left(t_{k}\right) & =\left[{\text{O}}_{2}\right]\left(t_{k}\right)+\zeta_{k},\;\zeta_{k}∼N\left(0,\sigma_{{\text{O}}_{2}}^{2}\left[{\text{O}}_{2}\right]\left(t_{k}\right)+0.01\right),\\ {}[{\text{CO}}_{2}{]}_{m}\left(t_{k}\right) & =\left[{\text{CO}}_{2}\right]\left(t_{k}\right)+\eta_{k},\;\eta_{k}∼N\left(0,\sigma_{{\text{CO}}_{2}}^{2}\left[{\text{CO}}_{2}\right]\left(t_{k}\right)+0.01\right). \end{array} \end{array}$$ (12)

Since the two gasses are measured with different sensors, we assume that the two noise terms are independent. We assume that the noise variances scale linearly with the gas concentrations, plus a small value to ensure positive definiteness of the *R* matrix in Eq (2). $\sigma_{{\text{O}}_{2}}^{2}$ and $\sigma_{{\text{CO}}_{2}}^{2}$ are nuisance parameters that must be estimated, such that the respiration and fermentation of pear fruit can be studied, but their precise estimation is not of direct interest to us. Since the variance of a gas concentration measurement must have the units of squared concentration, both $\sigma_{{\text{O}}_{2}}^{2}$ and $\sigma_{{\text{CO}}_{2}}^{2}$ must have units of concentration. Several sensor principles are available for measuring O_2_ concentrations in a practical setting, including gas chromatography, zirconium based sensors, paramagnetic sensors and fluorescence based optical sensors; CO_2_ concentrations can be measured using gas chromatography, infrared absorption and chemical gas sensors.

Before considering experimental design for this model, we first checked whether the model parameters can be correctly identified at all. This is because it is possible that two different model parameter values result in exactly the same output behavior, making it impossible to distinguish the true value of the model parameters from the data. We confirmed, using the STRIKE-GOLDD Matlab toolbox [32], that our respiration and fermentation model is structurally identifiable even with constant input levels, that do not change in time. Our piecewise constant input profiles form a super-set of the set of constant input profiles, which thus ensures that our experimental designs will result in an identifiable model.

### Prior information

Optimal experimental design for our non-linear respiration and fermentation model requires prior information, concerning the six respiration and fermentation parameters. Such prior information for pear respiration and fermentation can be found in [20]. We cannot directly utilize the published results, as [20] only report confidence intervals for each individual parameter, but no correlations between estimates. To deal with this problem, we reanalyzed 50 time series data-sets, each containing O_2_ and CO_2_ measurements from a single jar, made available to us by the authors, and which can be found in the S1 Table of this paper. We used a Bayesian data analysis technique to achieve this. More specifically, we utilized a Markov-chain Monte-Carlo method to re-estimate the parameters and quantify the uncertainty in the data [33]. The Markov-chain can be found in the S2 Table of this paper. The Markov-chain stores values sampled from the posterior distribution of the parameters. The chain can thus be utilized for numerically approximating the expectation in the robust criterion in Eq (7). In other words, we use the MCMC chain as an input to the expression in Eq (8).

Because the data of [20] were collected at different temperatures, our analysis took into account the effect of temperature on the maximal respiration and fermentation rate by means of the Arrhenius equations:

$$\begin{array}{c} \begin{array}{cc} V_{\text{m},{\text{O}}_{2}} & =V_{\text{m},{\text{O}}_{2},T_{r}}\text{exp}(\frac{E_{a,{\text{O}}_{2}}}{\bar{R}}(\frac{1}{T_{r}}-\frac{1}{T})),\\ V_{\text{m,f,}{\text{CO}}_{2}} & =V_{\text{m,f,}{\text{CO}}_{2},T_{r}}\text{exp}(\frac{E_{a,{\text{CO}}_{2}}}{\bar{R}}(\frac{1}{T_{r}}-\frac{1}{T})), \end{array} \end{array}$$ (13)

where $V_{\text{m},{\text{O}}_{2},T_{r}}$ and $V_{\text{m,f,}{\text{CO}}_{2},T_{r}}$ are the maximal respiration rates at a reference temperature *T*_*r*_ of 293.15 K, and $E_{a,{\text{O}}_{2}}$ and $E_{a,{\text{CO}}_{2}}$ are activation energies that describe how the reaction rates increase with the temperature *T*. These activation energies are nuisance parameters in our computation of optimal input profiles, as we only consider experiments at the reference temperature.

Another difference between the experiments of [20] and our experiments is that they used closed jars, instead of flow through experiments. Their system dynamics thus differ:

$$\begin{array}{c} \begin{array}{cc} V_{\text{j}}\frac{d\left[{\text{O}}_{2}\right]}{dt} & =-m_{\text{p}}r_{{\text{O}}_{2}}\left(t\right),\\ V_{\text{j}}\frac{d\left[{\text{CO}}_{2}\right]}{dt} & =m_{\text{p}}r_{{\text{CO}}_{2}}\left(t\right). \end{array} \end{array}$$ (14)

**Fig 2:**
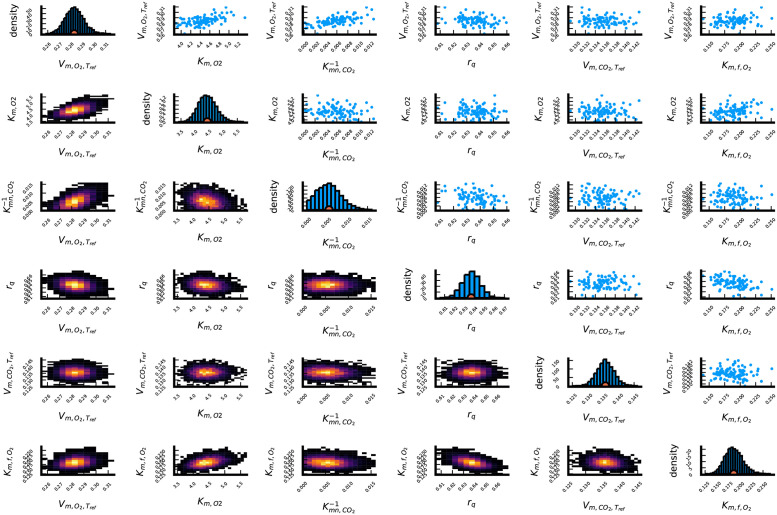
Summary of the posterior distribution resulting from a Bayesian data analysis of the data in [20] for the parameters of interest.

**Fig 3:**
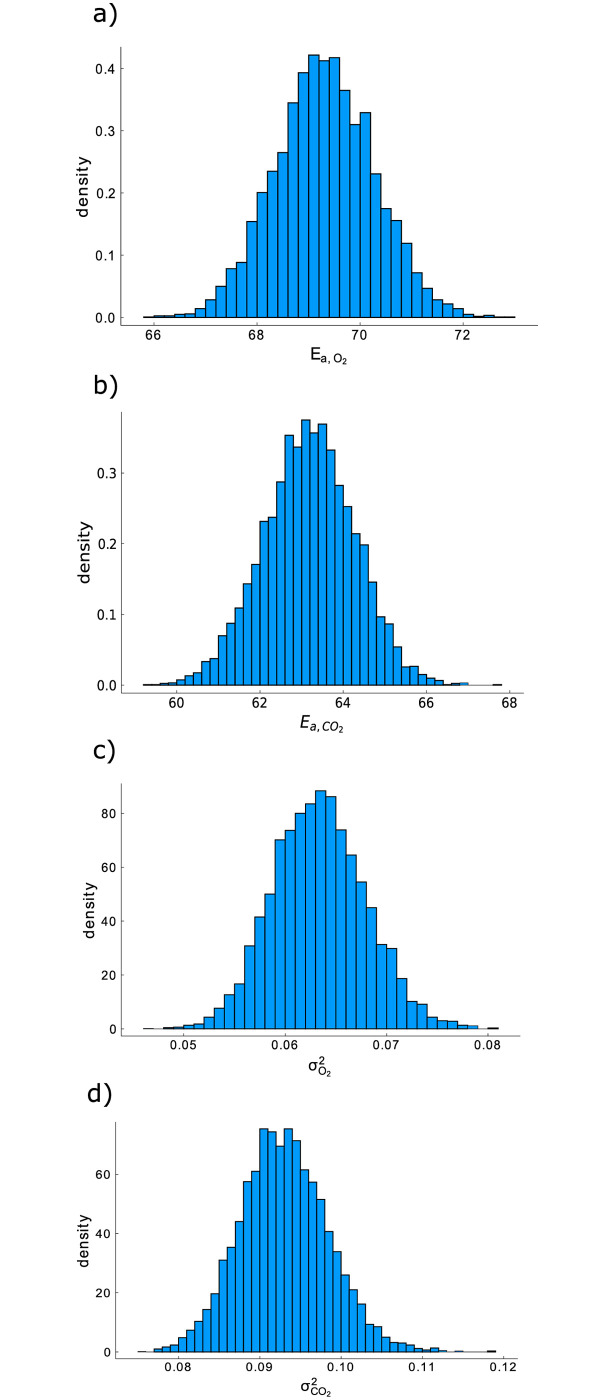
Summary of our Markov-chain Monte-Carlo analysis of the data in [20] for the nuisance parameters $E_{a,O_{2}}$, $E_{a,CO_{2}}$, $\sigma_{O_{2}}^{2}$ and $\sigma_{CO_{2}}^{2}$.

Fig 2 provides a summary of the results of our Bayesian analysis. On the diagonal of this figure, histograms of of the Markov-chain values of the six model parameters of interest ($V_{\text{m},{\text{O}}_{2}}$, $K_{\text{m},{\text{O}}_{2}}$, $K_{\text{mn},{\text{CO}}_{2}}$, *r*_q_, $V_{\text{m,f,}{\text{CO}}_{2}}$ and $K_{\text{m,f},{\text{O}}_{2}}$) are shown. The bullet at the horizontal axis of every histogram shows the average value for every parameter. Below the diagonal, two-dimensional heatmaps are shown, which visualize the correlation between the Markov-chain values for pairs of model parameters. The figures above the diagonal provide similar information, but show only 100 pairs of values from the Markov-chain. Histograms of the Markov-chain values for the nuisance parameters $E_{a,{\text{O}}_{2}}$, $E_{a,{\text{CO}}_{2}}$, $\sigma_{{\text{O}}_{2}}^{2}$ and $\sigma_{{\text{CO}}_{2}}^{2}$ are depicted in Fig 3. The estimation of the respiration inhibition parameter, $K_{\text{mn},{\text{CO}}_{2}}$, from the available data was problematic. For this reason, we had to reparametrize the model using the inverse of $K_{\text{mn},{\text{CO}}_{2}}$. As can be seen in the third histogram in Fig 2, $K_{\text{mn},{\text{CO}}_{2}}^{-1}$ takes values close to zero, which means $K_{\text{mn},{\text{CO}}_{2}}$ tends to infinity. The lack of information about $K_{\text{mn},{\text{CO}}_{2}}$ can be explained as follows: to precisely estimate this parameter, both high O_2_ and CO_2_ concentrations are needed. A high O_2_ concentration is required because otherwise there is no respiration that can be inhibited, and a high CO_2_ concentration is required because otherwise the inhibition has a negligible effect. Data points in which both gasses posses a high concentration do not occur in the data of [20]. The issues with the uncertainty about $K_{\text{mn},{\text{CO}}_{2}}$ were the catalyst for the development of our robust experimental design method. We found no other reliable way to quantify the uncertainty on this parameter, except working with a Markov-chain. $K_{\text{m,f},{\text{O}}_{2}}$ is the second hardest parameter to identify from the data of [20], because the O_2_ concentration needs to be in a specific range for this parameter to have an effect on the outputs. An O_2_ concentration much higher than $K_{\text{m,f},{\text{O}}_{2}}$ means no fermentation is happening at all, while an O_2_ concentration much lower than $K_{\text{m,f},{\text{O}}_{2}}$ implies maximal fermentation. The third most difficult to identify parameter is $K_{\text{m},{\text{O}}_{2}}$, because the model is only sensitive to this parameter when O_2_ concentrations are close to the value of this parameter.

### Markov-chain details

The data analysis was performed using the No U-Turn Sampling Markov-chain Monte-Carlo algorithm [34], as implemented in Turing.jl [35]. We ran 4 parallel Markov-chains, each starting from the maximum likelihood estimates of the parameters, and each chain comprises 1500 steps. We utilized truncated wide Gaussian distributions as prior distributions in the physically possible regions of the unknown parameters. For all parameters only positive values are possible, with *r*_q_ being at most 1. The means and standard deviations of these wide Gaussian distributions are found in Table 1. We used the posterior distribution resulting from our Bayesian analysis of the experiments in [20] as a prior distribution for the generation of the robust designs in this paper. However, as calculating robust optimal designs with the entire Markov-chain is numerically quite intensive, we discarded the first 500 step, and thinned the remaining 1000 by a factor 40. This gave us 100 samples to calculate the robust D-optimality criterion in Eq (7). These are also the 100 values shown in the top right half of Fig 2.

**Table 1:** Prior distribution for the Markov-chain Monte-Carlo analysis.

	$V_{\text{m},{\text{O}}_{2}}$	$K_{\text{m},{\text{O}}_{2}}$	$K_{\text{mn},{\text{CO}}_{2}}^{-1}$	*r*_q_	$V_{\text{m,f,}{\text{CO}}_{2}}$	$K_{\text{m,f},{\text{O}}_{2}}$	$E_{a,{\text{O}}_{2}}$	$E_{a,{\text{CO}}_{2}}$	$\sigma_{{\text{O}}_{2}}^{2}$	$\sigma_{{\text{CO}}_{2}}^{2}$
units	$\frac{\mu \text{mol}}{\text{kg}\;\text{s}}$	kPa	$\frac{1}{\text{kPa}}$	-	$\frac{\mu \text{mol}}{\text{kg}\;\text{s}}$	kPa	$\frac{\text{kJ}}{\text{mol}}$	$\frac{\text{kJ}}{\text{mol}}$	$\frac{\mu \text{mol}}{{\text{m}}^{3}}$	$\frac{\mu \text{mol}}{{\text{m}}^{3}}$
mean	0.3	5	0.005	0.6	0.1	0.2	70	60	0.03	0.07
std	0.3/3	5/3	0.005	0.6/3	0.1/3	0.2/3	70/3	60/3	0.03/3	0.07/3

## Results

In our examples, we consider experiments lasting 24 h with measurements taken every minute, i.e. *t*_*e*_ = 24 h and *N* = 1440. The maximum and minimum flow rates are equal to 4 l h^−1^ and 0.1 l h^−1^, respectively. The input gas concentrations are allowed to vary between 0 kPa and 21 kPa. We did not use a minimum flow rate of 0 l h^−1^, because at zero flow there is no difference in system response between a maximal or minimal gas input concentration. This implies that the design selection criteria from Eqs (6) and (8) are flat in certain directions, causing numerical issues for gradient based optimizers. Working with a strictly positive minimum flow rate avoids this issue. We also tried remedying this issue using gradient free optimizers, such as the subplex method and the Nelder-Mead method [36, 37]. However, these methods did not converge for our robust experimental design methodology, even after 36 hours of computation time, nor did they find a better robust design than the gradient based methods during that time. All computations were run on an Intel Core i7–6700K CPU @ 4.00GHz.

### Locally optimal designs for the respiration and fermentation model

**Fig 4:**
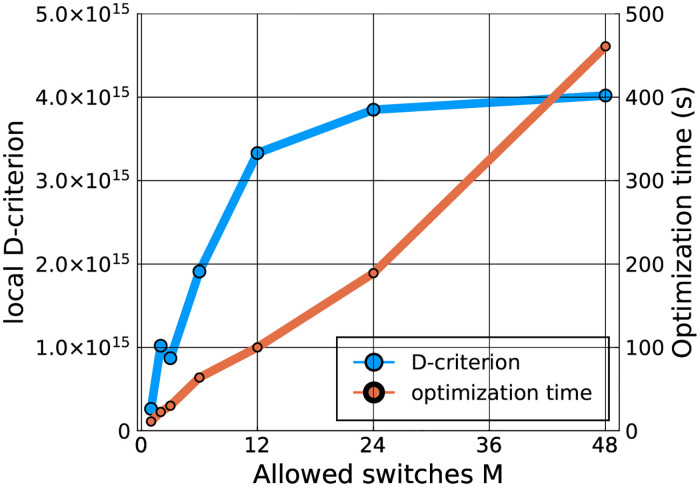
Convergence of the local D-optimality criterion and required computing time for finer discretizations of the controls.

We take the average values of the Markov-chains of the parameters of interest, as well as the average of the Markov-chain values of the parameters $\sigma_{{\text{O}}_{2}}^{2}$ and $\sigma_{{\text{CO}}_{2}}^{2}$ to evaluate the local D-criterion in Eq (7). These values are shown in Table 2, and indicated by a bullet in the histograms on the diagonal of Fig 2. We start by analyzing the effect of an increasing refinement of the discretization of the inputs ***u***(*t*). In Fig 4, which shows the local D-criterion value as a function of the number of times *M* the input signal is allowed to switch, we see that the D-criterion no longer improves noticeably after *M* = 24, which corresponds to a switch every hour. The experimental design obtained with *M* = 12, which corresponds to a switch every two hours, already performs well. Therefore, in the remainder of this work, we consider O_2_, CO_2_ and flow rate inputs that remain constant for two-hour time periods. The computational time required to optimize the designs are also shown in Fig 4. The optimization time increases linearly as the discretization becomes finer, but, even for *M* = 48, the optimization of the locally optimal design only requires 7 minutes.

**Table 2:** Parameter values used for the optimization of the locally optimal designs.

$V_{\text{m},{\text{O}}_{2}}$	$K_{\text{m},{\text{O}}_{2}}$	$K_{\text{mn},{\text{CO}}_{2}}$	*r*_q_	$V_{\text{m,f,}{\text{CO}}_{2}}$	$K_{\text{m,f},{\text{O}}_{2}}$	$\sigma_{{\text{O}}_{2}}^{2}$	$\sigma_{{\text{CO}}_{2}}^{2}$
μmol kg^−1^ s^−1^	kPa	kPa	-	μmol kg^−1^ s^−1^	kPa	μmol m^−3^	μmol m^−3^
0.283	4.43	182.5	0.637	0.136	0.187	0.063	0.093

**Fig 5:**
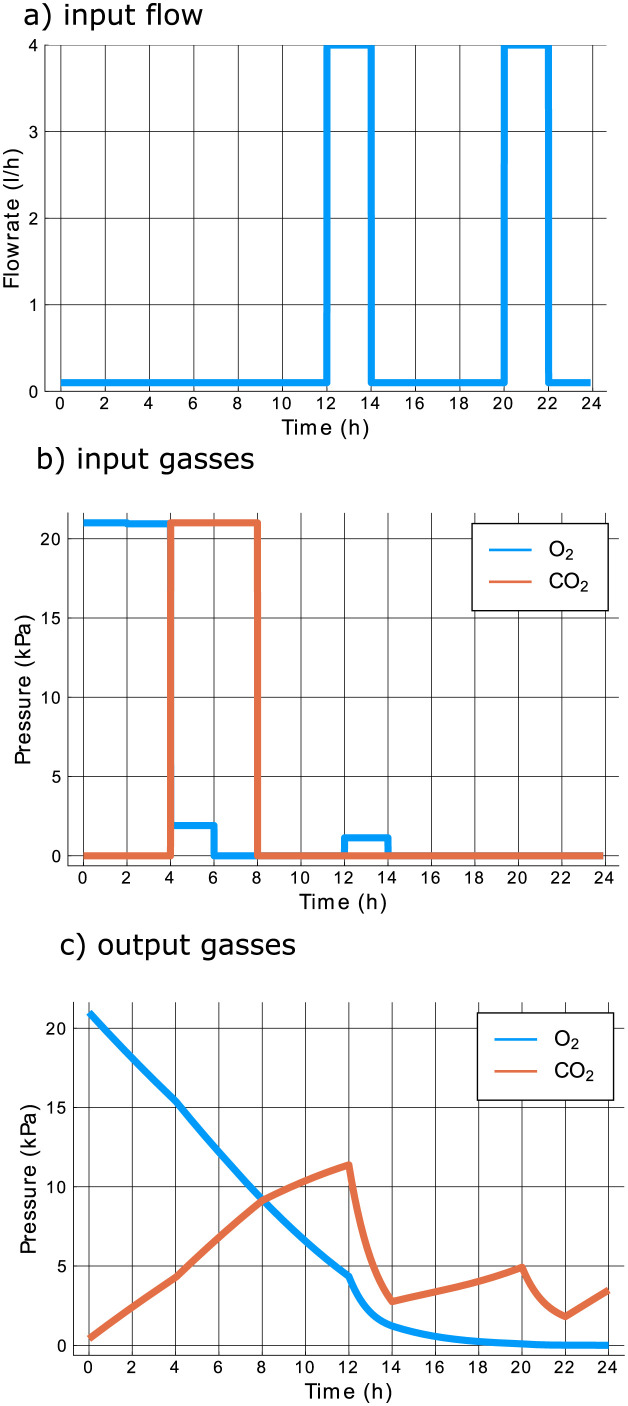
Locally optimal experiment and simulated output, with control input switches every two hours.

The locally optimal design for the scenario in which the inputs are allowed to vary every two hours is shown in detail in Fig 5a and 5b, together with the simulated outputs in Fig 5c, evaluated at the values of the model parameters used to optimize the design. During the first half of the experiment, no pumping actions occur, which causes the CO_2_ concentration to rise inside the jar. However, the gas concentrations cannot remain high throughout the entire experiment, since the O_2_ concentration must be low to learn about non-saturated respiration, and fermentation. The CO_2_ concentration is not allowed to run up too high, as this compromises the ability to precisely determine the fermentation parameters. This can be intuitively understood by considering the system in an extreme scenario where the atmosphere in the jar consists entirely of CO_2_. In this scenario, the outflow will also be pure CO_2_ regardless of the values of the fermentation parameters. Information about the fermentation parameters is then only incorporated in the outflow rate, but this is not a measured output. This illustrates how optimal experiments automatically take into account the specifics of the measurement setup. The importance of low CO_2_ concentrations is the reason why air, with almost zero O_2_ and CO_2_ inlet concentrations is pumped into the jar in the interval between 12 h and 14 h. The pumping action at 20 h, again involving zero O_2_ and CO_2_ concentrations, is performed for similar reasons.

**Fig 6:**
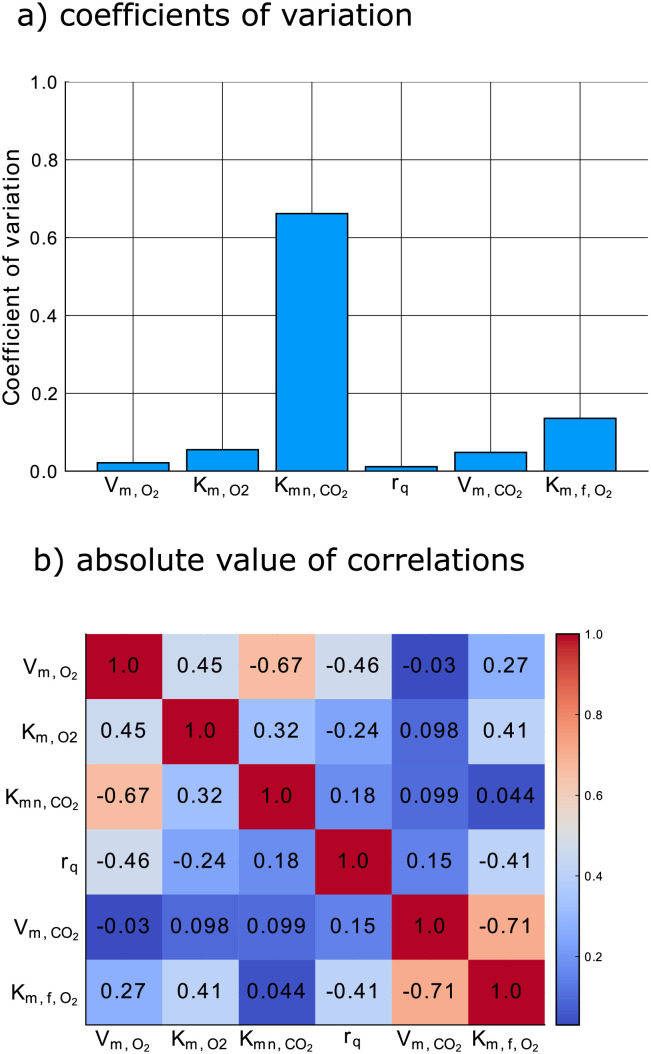
Summary of the information gained from the locally optimal experiment in Fig 5.

Fig 6a shows the coefficients of variation of all six model parameters, defined as the ratio of the standard errors, as given by the diagonal elements of the inverse of the FIM, and the model parameter values used for the generation of the local optimal design. The three inhibition parameters ($K_{\text{m},{\text{O}}_{2}}$, $K_{\text{mn},{\text{CO}}_{2}}$ and $K_{\text{m,f},{\text{O}}_{2}}$) remain the most difficult to estimate ones. Now, however, in our experiment involving a single jar, instead of using the data from 50 jars, made available to us by [20], the model parameters can be estimated much more precisely.

**Fig 7:**
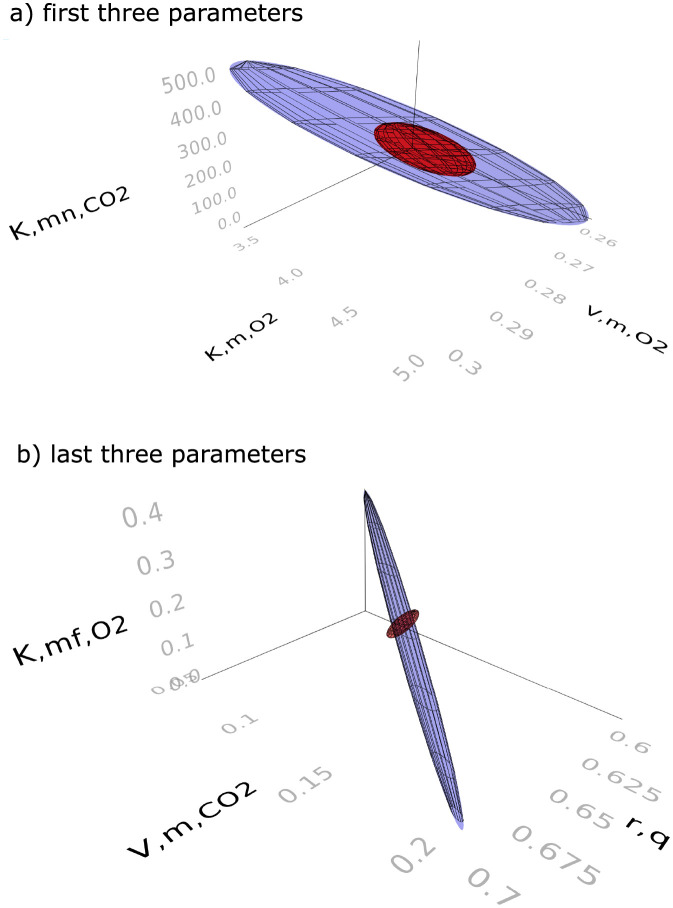
95% confidence ellipsoids comparing the locally optimal experimental design (red) and the heuristic experimental design technique from [20] (blue). a. first three parameters ($V_{\text{m},{\text{O}}_{2}}$, $K_{\text{m},{\text{O}}_{2}}$, $K_{\text{mn},{\text{CO}}_{2}}$) b. last three parameters (*r*_q_, *V*_m,f,_, $K_{\text{m,f},{\text{O}}_{2}}$).

Fig 6b shows a map of the absolute values of the correlations of the estimates that would be obtained if the locally optimal experiment were to be used. Because there are no strong correlations between the first three parameters and the last three parameters, we can thus visualize the FIM in Eq (2) using two ellipsoids. The axes of the first ellipsoid are in the directions of the eigenvectors of the inverse of the top left quarter (a 3 by 3 matrix) of the FIM, and the lengths of the axes are the corresponding eigenvalues. The second ellipsoid is similarly based on the bottom right quarter. More specifically, we can use one ellipsoid to show the gain in information for the parameters $V_{\text{m},{\text{O}}_{2}}$, $K_{\text{m},{\text{O}}_{2}}$, and $K_{\text{mn},{\text{CO}}_{2}}$ and another ellipsoid to show the gain in information for the parameters r_q_, $V_{\text{m,f,}{\text{CO}}_{2}}$ and $K_{\text{m,f,}{\text{O}}_{2}}$. These ellipsoids are shown in red in Fig 7a and 7b for the experiment allowing changes in inputs every two hours, and are compared to the ellipsoids in blue resulting from the heuristic experimentation technique used by [20] with the volume of the jar, mass of pears, initial conditions, sampling times and total experimentation time equalized between the two methods. The volume of the ellipsoids of the locally optimal experiment are smaller.

### Robust optimal designs for the respiration and fermentation model

**Fig 8:**
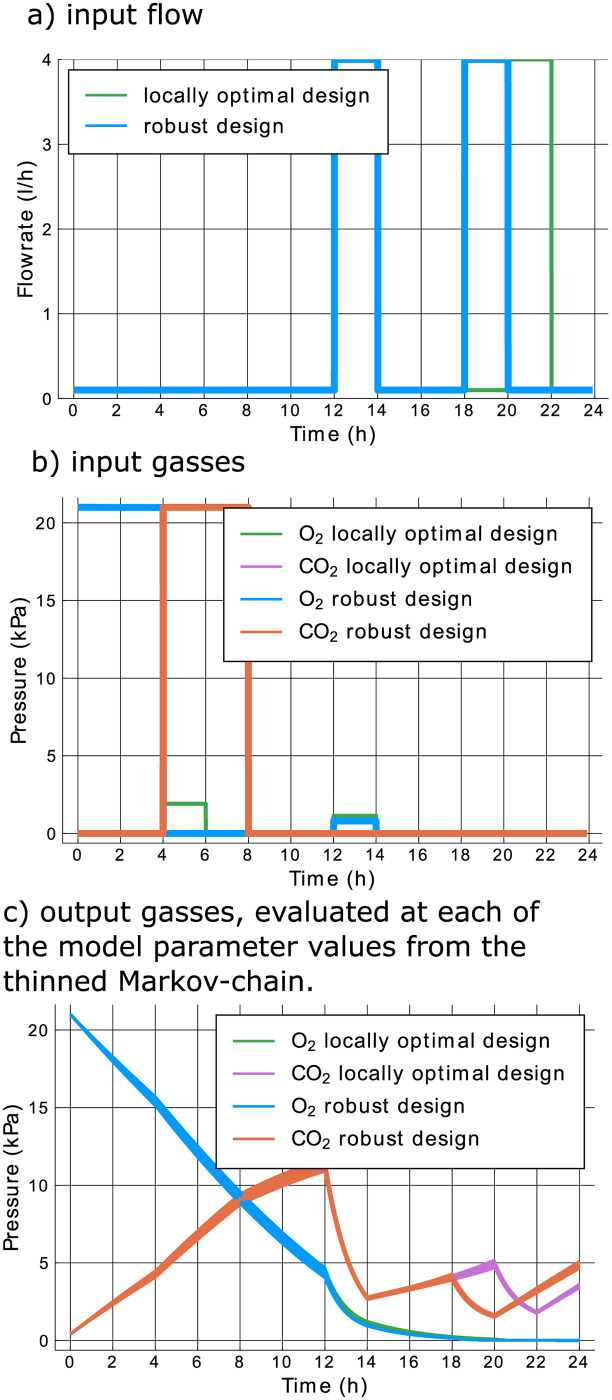
Robust optimal experiment and simulated output, obtained using 100 parameter values from the Markov-chain and control input switching every two hours.

**Fig 9:**
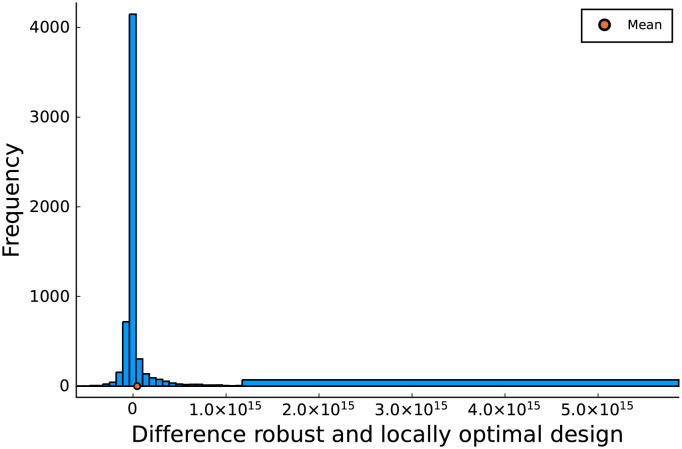
Histogram of difference in determinants of the FIM of the robust and locally optimal designs for 6000 parameters from the prior distribution. Positive values mean the robust design performs better. Note that the most right bin of the histogram spans a wider interval than other bins. This is done to better showcase the heavy right tail of the histogram.

We now continue by searching a more robust design than the locally optimal design in Fig 5. Instead of optimizing the determinant of the FIM in Eq (2) for the means of the Markov-chain values, we optimize the mean determinant for a thinned version of the Markov-chain. These 100 values from the thinned chain are graphically shown by the blue bullets in the upper right hand part of Fig 2. The design found by maximizing the robust optimality criterion in Eq (8) is shown in Fig 8a and 8b. The simulated output for all values of the thinned Markov-chain is shown in Fig 8c. The robust design exhibits several similarities to the locally optimal design, with one key difference: the second pumping action occurs two hours earlier in the robust design. Fig 9 shows the difference in performance between the robust and the locally optimal experiment. More specifically, the histogram shows the difference between the determinant of the FIM in Eq (2) for both the robust and locally optimal design at all 6000 model parameter values from four parallel Markov-chains. For many possible model parameter values, the local and robust design perform almost equally well. However, the histogram clearly has a heavy right tail, showing that, for some parameter values, the robust design performs significantly better. As the histogram does not posses a heavy left tail, the reverse is not true. The robust design is thus substantially less sensitive to the exact values of the model parameters. It therefore provides a better guarantee for a highly informative experiment than the locally optimal design. Since this result holds for the entire Markov-chain and not just the thinned version we can be confident that the thinned version is sufficient to summarize the prior uncertainty, and that the resulting robust design does not only work well for the specific model parameter values used to evaluate the optimality criterion in Eq (8), but also for parameter values other than the 100 values used in this equation. The heavy right tail consists of those elements of the Markov-chain which have a low value for the hard to estimate parameter $K_{\text{mn},{\text{CO}}_{2}}$. For low values of this parameter, the parameter is best estimated at lower CO_2_ concentrations in the jar, while there is also still enough O_2_ left for the inhibition effect on respiration to be noticeable. This explains the earlier pump in the robust experiment. Finally, the positive mean value for the difference in determinants, indicated by the orange dot in Fig 9, proves that the robust design is expected to do better for the region of prior uncertainty. We also calculated the locally optimal design for one of the model parameter values out of the right tail of Fig 9 and found that the locally optimal design for this parameter value was identical to the robust design. So, it seems that the robust design is influenced heavily by a few parameter values. However, for the parameter value for which the locally optimal design in Fig 5 performs the best, the robust design does not perform much worse, relative to the difference in performance between those two designs for model parameter values in the tail of Fig 8. To summarize: the robust design does significantly better for a few of the possible parameter values and only slightly worse for the majority of possible parameter values.

In Table 3, we also compare, using the full Markov-chain, our robust experimental design method based on a thinned Markov-chain, which can approximate arbitrary distributions, to the robust experimental design method of [12], which uses 13 sigma-points to summarize the uncertainty on the model parameters, and is thus computationally less intensive. These sigma-points can be found in the S3 Table of this paper. This method requires a mean and covariance matrix summarizing the prior uncertainty on the model parameters to calculate the sigma-points. We used the mean and covariance matrix our Markov-chain for this purpose. For the hard to estimate parameter $K_{\text{mn},{\text{CO}}_{2}}$, we again reparametrized the model using the inverse, $K_{\text{mn},{\text{CO}}_{2}}^{-1}$. We followed the recommendation of [12] regarding the weights of the sigma-points. The sigma-point method results in a design that performs slightly worse than the locally optimal design, showing that sigma-points do not always provide good summary of the uncertainty in the model parameters. The inputs of the sigma-point based design are very similar to the locally optimal design. They only very slightly differ in the gas inputs between 4–8h. Table 3 also shows the computing times for the locally optimal design, the design based on the Markov-chain, and the design based on the sigma-point method. The robust design requires a computational time that is roughly 100 times longer than that of than the locally optimal design and 10 times longer than that of the design based on the sigma-point method.

**Table 3:** Parameters used for the optimization of the construction of the locally optimal designs.

	Markov-chain Monte-Carlo optimal design	locally optimal design	sigma point optimal design
Robust D-criterion	1.07×10^16^	1.05×10^16^	1.04×10^16^
Optimization time (s)	11266	100	1430

## Discussion and future work

### Alternative design selection criteria

In this paper, we presented a robust experimental design methodology for dynamic models and applied that methodology to precisely estimate the respiration and fermentation parameters of fruit and vegetables. We achieved this by quantifying the information content of the experiments using the determinant of the Fisher information matrix averaged over a prior distribution, represented by a Markov-chain. However, the Fisher information matrix is only an approximation of the inverse of the model parameter covariance matrix. Another approach to approximate the covariance matrix based on simulating multiple possible data-sets, and estimating the model parameters from each data-set is suggested by [38]. This approach is numerically much more intensive than an approach based on the FIM, and is therefore infeasible in the context of optimal experimental design. Another promising approach would be to quantify information based on the expected Kullback-Leibler divergence (KL-div) between the prior and posterior distributions [39]. For experiments with a large number of observations, the expected KL-div is asymptotically equal to our robust D-optimality criterion. For a small number of measurements, the KL-div approach might be superior as it does not utilize normal approximations. One major downside of using the KL-div is its computational complexity, as it involves calculating a high dimensional integral over all possible outcomes ***y*** of the experiment. This is generally done using a double loop Monte-Carlo integration method [40]. Some research has been done using this method for determining optimal sampling times of dynamic systems [41], but this work does not consider optimal control. Therefore, the dynamic system in [41] does not need to be solved repeatedly for different control actions. Instead, only a single dynamic system needs to be solved that can be evaluated at different possible sampling times. Selection of input profiles based on the KL-div is considered in [42], but only for a small discrete set of possible input profiles. In contrast, in our paper, we optimize the experimental design over a large continuous space of possible experimental designs. Recently, advances have been made in lowering the computational burden of the KL-div based approach by considering surrogate functions that approximate the KL-div based on polynomial chaos expansions [43]. Another approach to lower the computational burden is based on variational Bayesian techniques [44], where the inner Monte-Carlo loop is replaced by optimizing a variational distribution. This technique then allows for jointly optimizing the design and variational parameters [45]. In future work, we will employ these variational Bayesian techniques for adaptive dynamic experiments, where the design is modified online as data is collected.

### Model uncertainty

Throughout this paper, we have assumed that the model structure has been identified correctly, and that only the model parameters must be estimated. However, often there is also substantial uncertainty about which model structure is correct. An experiment (or sequence of experiments) must then be constructed such that a precise determination of both the model structure and the model parameters is ensured. For robust experimental design, this involves constructing a joint prior distribution over the possible model structures and their model parameters. For the techniques used in this paper, constructing such a joint prior would be difficult, since the Markov-chain Monte-Carlo methods we employed, such as NUTS, can only deal with continuous parameters, and not discrete ones like different possible model structures [33]. Possibly, this issue could be overcome using Gibbs sampling [35]. We also assumed that the measurement noise was heteroscedastically normally distributed. Since the gas concentrations should always be positive, other distributions, such as the log-normal distributions might also be considered. For these distributions, however, the definition of the Fisher information matrix in Eq (2) would have to be generalized [2], which would lead to much more complex optimization problems.

### State discretization

In this work, we discretized only the controls ***u***, but not the states ***x***. The discretization of states is applied using multiple shooting and collocation based dynamic optimization approaches [46]. Generally, these methods lead to optimization problems that are faster to solve, but which might result in poorer designs. Besides the faster computing time, one additional reason to consider discretization of the states would be the presence of additional constraints on the states, which our problem does not contain. This is because violations of such constraints are difficult to check without a discretization, and adding these discretized states as variables to the optimization problem. In our work, the dynamic states are integrated away by the differential equation solver. We could still use the solution of the differential equation to check state violations and add a term to the optimization objective that punishes such violations. A more detailed discussion on the incorporation of constraints into optimal dynamic experiments is provided by [13].

### Reverse automatic differentiation

We used forward mode automatic differentiation to calculate the sensitivities to the unknown parameters ***θ***, required to obtain the FIM in Eq (2), and this was further nested to calculate the gradient of the D-criteria in Eqs (6) and (8) with respect to the control parameters ***u***. Forward mode automatic differentiation generally performs well for functions with a small number of inputs, relative to the number of outputs. For our respiration model, it thus was a good choice for calculating the unknown parameter sensitivities present in the FIM. Reverse mode automatic differentiation performs better for functions with many more inputs than outputs. It thus seems like a natural choice to calculate the gradients necessary for the optimization of the control parameters. However, there is currently not yet a mature implementation of reverse over forward mode automatic differentiation in the Julia ecosystem [27].

## Supporting information

S1 Table Pear respiration and fermentation data. (ZIP)

S2 Table Markov-chain summarizing model parameter uncertainty. (ZIP)

S3 Table Sigma-points summarizing model parameter uncertainty. (ZIP)
